# Profile of urinary amino acids and their post-translational modifications (PTM) including advanced glycation end-products (AGEs) of lysine, arginine and cysteine in lean and obese ZSF1 rats

**DOI:** 10.1007/s00726-021-03042-3

**Published:** 2021-07-11

**Authors:** Svetlana Baskal, Petra Büttner, Sarah Werner, Christian Besler, Philipp Lurz, Holger Thiele, Dimitrios Tsikas

**Affiliations:** 1 Institute of Toxicology, Hannover Medical School, Core Unit Proteomics, Carl-Neuberg-Strasse 1, 30623 Hannover, Germany; 2 Department of Cardiology, Heart Center Leipzig at University Leipzig, Leipzig, Germany

**Keywords:** AGEs, Amino acids, Glycation, HFpEF, Methylation, Obesity, PTM, Urine, ZSF1 rat

## Abstract

Heart failure with preserved ejection fraction (HFpEF) is associated with high mortality and has an increasing prevalence associated with the demographic change and limited therapeutic options. Underlying mechanisms are largely elusive and need to be explored to identify specific biomarkers and new targets, which mirror disease progression and intervention success. Obese ZSF1 (O-ZSF1) rats are a useful animal model, as they spontaneously develop hypertension, hyperlipidemia and glucose intolerance and finally HFpEF. The urinary profile of amino acids and their metabolites of post-translational modifications (PTM), including the advanced glycation end-products (AGEs) of lysine, arginine and cysteine, are poorly investigated in HFpEF and ZSF1 rats. The aim of the present study was to characterize the status of free amino acids and their metabolites of PTM and glycation in lean ZSF1 (L-ZSF1) and O-ZSF1 rats in urine aiming to find possible effects of glucose on the excretion of native and modified amino acids. In the urine of twelve L-ZSF1 and twelve O-ZFS1 rats collected at the age of 20 weeks, we measured the concentration of native and modified amino acids by reliable previously validated stable-isotope dilution gas chromatography-mass spectrometry (GC–MS) approaches. Serum glucose was 1.39-fold higher in the O-ZSF1 rats, while urinary creatinine concentration was 2.5-fold lower in the O-ZSF1 rats. We observed many differences in urinary amino acids excretion between L-ZSF1 and O-ZSF1 rats. The creatinine-corrected homoarginine excretion was twofold lower in the O-ZSF1 rats. We also observed distinct associations between the concentrations of serum glucose and urinary amino acids including their PTM and AGE metabolites in the L-ZSF1 and O-ZSF1 rats. Our study shows that PTM metabolites and AGEs are consistently lower in the L-ZSF1 than in the O-ZSF1 rats. Serum malondialdehyde (MDA) concentration was higher in the O-ZSF1 rats. These results suggest that hyperglycemia, hyperlipidemia and elevated oxidative stress in the O-ZSF1 rats favor PTM methylation of arginine and lysine and the glycation of lysine and cysteine. The area under the receiver operation characteristic (ROC) curve values were 0.996 for serum glucose, 0.951 for urinary creatinine, 0.939 for serum MDA, 0.885 for *N*^*ε*^-carboxyethyl-lysine, 0.830 for carboxyethyl-cysteine, and 0.792 for monomethyl-lysine. Non-invasive measurement of methylation and glycation products of arginine, lysine and cysteine residues in proteins in urine of L-ZSF1 and O-ZSF1 rats may be useful in studying pathophysiology and pharmacology of HFpEF.

## Introduction

Heart failure with preserved ejection fraction (HFpEF) is associated with high morbidity and mortality (From and Borlaug 2011). While systolic function in HFpEF is normal, patients suffer from diastolic dysfunction, dyspnea and exercise intolerance (From and Borlaug 2011; Maeder and Rickli 2013). Currently, there are no evidence-based therapies for HFpEF (From and Borlaug 2011; Tschöpe et al. 2019). Limited knowledge of underlying pathomechanisms hampers the development of new approaches. Importantly, it was found that arterial stiffness on the left ventricle and impaired endothelial function are involved in HFpEF (Baldassarri et al. 2018). Both conditions are associated with an imbalance in the synthesis and metabolism of nitric oxide (NO), a potent endogenous vasodilator (Schiattarella et al. 2019). NO is synthesized by the NO synthases (NOS)-catalyzed oxidation of one nitrogen atom of the guanidine group of l-arginine (Arg), with l-citrulline being the second oxidation product. NOS activity is dependent upon the availability of the physiological substrates Arg and l-homoarginine (hArg) and is inhibited by asymmetric dimethylarginine (ADMA) (Schiattarella et al. 2019). Importantly, ADMA is a major product of post-translational modification (PTM) of certain Arg residues in proteins (Tsikas 2020). Mortality in HFpEF is associated with low circulating hArg concentrations (Atzler et al. 2013). Further, low Arg and high ADMA concentrations were found to be associated with typical pathophysiological functional alterations of the heart in HFpEF patients (Baldassarri et al. 2018).

PTM of Arg, l-lysine (Lys) and l-cysteine (Cys) residues in proteins includes methylation and glycation (Arg, Lys, Cys) and citrullination (Arg). Glycation is a particular PTM that leads to generation of amino acid metabolites that are widely known as advanced glycation end-products (AGEs) (reviewed by Nagai et al. 2014; Anwar et al. 2021; Neff and Bradshaw 2021). Glucose, fructose, trioses, and dicarbonyl compounds, such as glyoxal and methylglyoxal, are glycation initiators. PTM metabolites in general and AGEs in particular are considered to be associated with elevated oxidative stress mainly due to hyperglycemia and to exert detrimental effects largely by not yet well-understood mechanisms (Nagai et al. 2014). The status of AGEs in plasma of patients with HFpEF has been sporadically reported (Willemsen et al. 2012; for review see Paulus and Dal Canto 2018). Because the underlying mechanisms of the advanced glycation of Arg, Lys and Cys are elusive, we were interested to close these gaps. For our investigations, we used the ZSF1 rat model, a proven model of human HFpEF that is considered to fulfill virtually all required criteria of this disease (Valero-Munoz et al. 2017).

Obese ZSF1 (O-ZSF1) rats are a F1 hybrid cross-breed from spontaneous hypertensive rats and Zucker diabetes rats. O-ZSF1 rats spontaneously develop hypertension, hyperlipidemia, glucose intolerance, exercise intolerance (Bowen et al. 2017) and finally HFpEF. However, it is unknown whether advanced glycation of Arg, Lys and Cys also plays a role in HFpEF. The aim of the present study was to characterize the status of glycation and oxidative stress in O-ZSF1 and in healthy lean ZSF1 (L-ZSF1) rats serving as controls. To achieve this goal, we measured the urinary amino acids profile and representative PTM metabolites and AGEs of Arg, Lys and Cys, as well as malondialdehyde (MDA), an established biomarker of lipid peroxidation (Tsikas 2017).

## Materials and methods

### ZSF1 rats

ZSF1 hybrid rats crossed between a Zucker diabetes fatty female and a spontaneous hypertensive heart failure male rat were used as an animal model of HFpEF (ZSF1-*Lepr*^*fa*^* Lepr*^*cp*^*/Crl* Hybrid, Charles River, Indianapolis, USA). Female O-ZSF1 rats (*n* = 12) rats at 20 weeks of age were compared with lean strain-matched ZSF1 rats (*n* = 12). All animals were kept at identical conditions under a 12:12 h-light/dark cycle with food and water provided ad libitum. Standard chow rich in energy and protein content was delivered by Ssniff (Soest, Germany). Body weight and food intake were recorded every week. Urine samples were collected and stored at − 80 °C until analysis. All experiments and procedures were performed in accordance with relevant guidelines and regulations and were approved by the local Animal Research Council, University of Leipzig, and the Landesbehörde Sachsen (TVV 30/18).

### Chemicals and reagents

All synthetic amino acids and derivatization reagents were of analytical grade and were commercially obtained from various manufacturers in Germany (Sigma-Aldrich, Merck, Cayman, Iris Biotech GmbH, Carbosynth, TCI, Chemsolute). Glassware for GC–MS (1.5-mL auto-sampler vials and 0.2-mL microvials) and the fused-silica capillary column Optima 17 (15 m × 0.25 mm I.D., 0.25-µm film thickness) were purchased from Macherey–Nagel (Düren, Germany). Separate stock solutions of amino acids were prepared by dissolving accurately weighed amounts of commercially available unlabeled and stable-isotope labeled AGEs in deionized water. Stock solutions were diluted with deionized water as appropriate.

### GC–MS analyses of urinary creatinine, amino acids and their metabolites

Amino acids and their metabolites including creatinine were quantitated by GC–MS using their stable-isotope-labeled analogs as internal standards. All analyses were performed under routine conditions on a GC–MS apparatus consisting of a single quadrupole mass spectrometer model ISQ, a Trace 1210 series gas chromatograph and an AS1310 auto-sampler from ThermoFisher (Dreieich, Germany). The oven temperature was held at 40 °C for 0.5 min and ramped to 210 °C at a rate of 15 °C/min and then to 320 °C at a rate of 35 °C/min. Interface and ion-source temperatures were set to 300 °C and 250 °C, respectively. Electron energy was 70 eV and electron current 50 µA. Methane was used as the reagent gas for negative-ion chemical ionization at a constant flow rate of 2.4 mL/min. In quantitative analyses, the dwell time was 50 ms or 100 ms for each ion in the selected-ion monitoring (SIM) mode and the electron multiplier voltage was set to 1400 V.

**Fig. 1:**
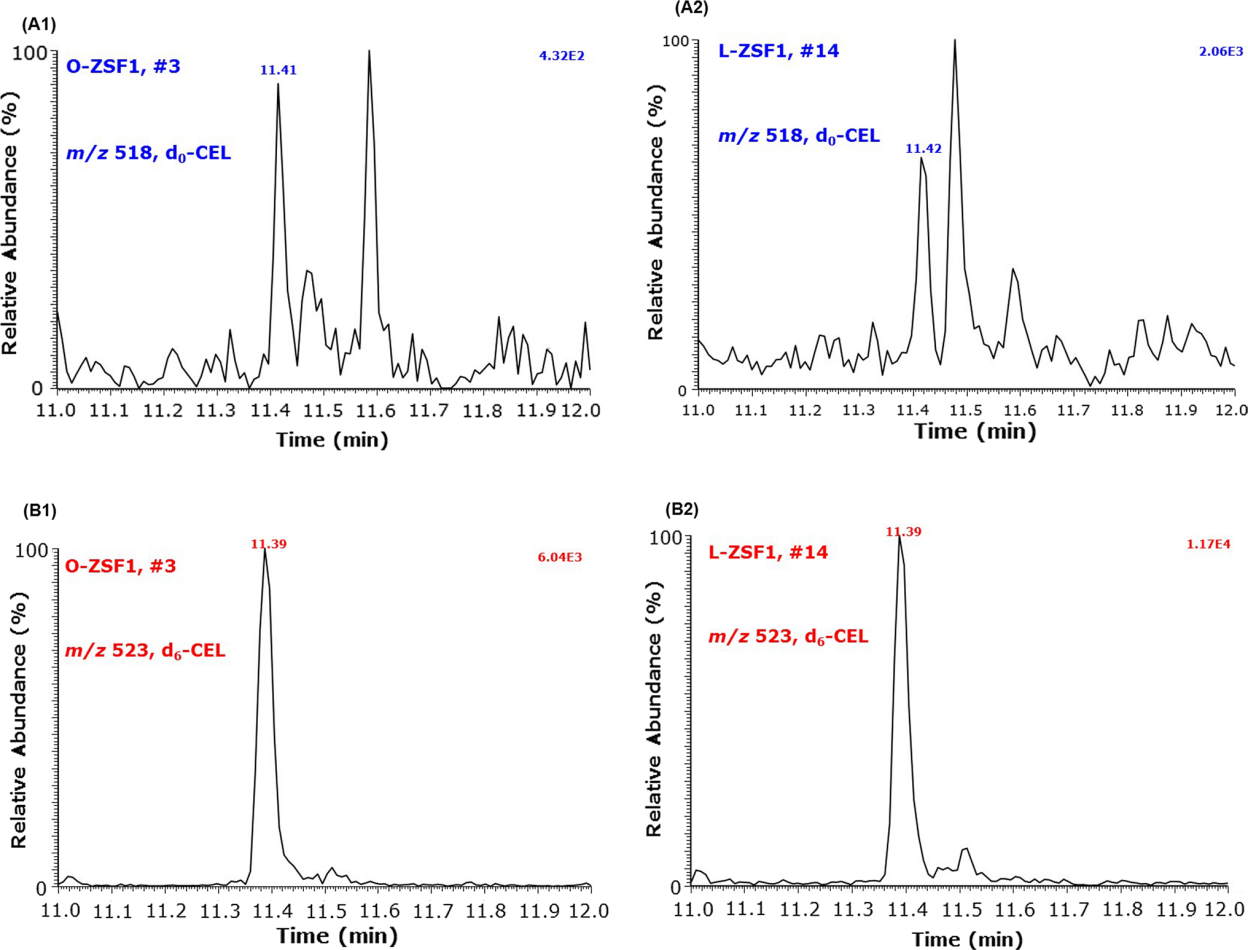
Representative chromatograms from GC–MS analyses of *N*^*ε*^-carboxyethyl-lysine (CEL) in urine samples of an O-ZSF1 rat (left panel, #3) and an L-ZSF1 rat (right panel, #14). SIM of *m/z* 518 for endogenous d_0_-CEL (upper tracings) and *m/z* 523 for the internal standard d_6_-CEL (lower tracings) was performed (see Baskal et al. 2021). Retention time: 11.4 min for unlabeled CEL (d_o_-CEL); 11.39 min for deuterium-labeled CEL (d_6_-CEL)

Urinary amino acids and their PTM and glycation metabolites were measured in 10-µL aliquots of native urine as described previously for native amino acids (Hanff et al. 2019; Baskal et al. 2021). Representative GC–MS chromatograms for *N*^*ε*^-carboxyethyl-lysine (CEL) are shown in Fig. 1. Dimethylamine (DMA), the major metabolite of ADMA (Tsikas 2020), was measured in urine by GC–MS as described elsewhere (Tsikas et al. 2007). Malondialdehyde (MDA), a biomarker of lipid peroxidation (Tsikas 2017), was measured in serum by GC–MS (Tsikas et al. 2016). Urinary creatinine was measured in 10 µL of 1:10 (v/v) urine samples diluted with 90-µL deionized water as reported elsewhere (Tsikas et al. 2010). The concentration (in µM) of the analytes in the urine was divided by the respective concentration of creatinine (in mM) and the result is expressed as µM analyte per mM creatinine (i.e., µM/mM).

### Statistical analyses and data presentation

Results are reported as mean with standard deviation for normally distributed and as median with interquartile range for non-normally distributed data. Comparisons between groups were done using Mann–Whitney *U* test or Kruskal–Wallis *t* test. A *P* value of 0.05 was considered significant. Correlations were performed after Spearman (S) or Pearson (P) as specified in text. GraphPad Prism 7 (GraphPad Software, San Diego, USA) was used in statistical analyses and to prepare graphs including the area under the ROC (AUROC) curves.

## Results

Table 1 summarizes the characteristics of the investigated rats. O-ZSF1 rats had a 50% higher food intake than L-ZSF1. At sacrifice at an age of 20 weeks, O-ZSF1 rats had significantly higher body weight as well as heavier heart, liver and kidney. Serum glucose was higher (on average 1.39-fold) in the O-ZSF1 compared to the L-ZSF1 rats. Urinary creatinine concentration was lower (on average 2.5-fold) in O-ZSF1 compared to L-ZSF1. The creatinine-corrected urinary excretion rates of the investigated amino acids and their PTM and glycation metabolites in the two groups are summarized in Table 2.

**Table 1:** Characteristics of the O-ZSF1 (*n* = 12) and L-ZSF1 (*n* = 12) rats of the study at 20 weeks of age

Parameter/rat group	O-ZSF1	L-ZSF1	*P* value^a^
Serum glucose (mM)	31.2 ± 1.3	22.4 ± 3.1	< 0.0001
Urine creatinine (mM)	0.79 [0.67–1.01]	1.98 [1.61–2.57]	< 0.0001
Food intake (g/day)	23 ± 1.5	15 ± 1.3	< 0.0001
Body weight (g)	468 ± 24	235 ± 9	< 0.0001
Heart weight (g)	1.38 ± 0.07	0.93 ± 0.05	< 0.0001
Kidney weight (g)	1.54 ± 0.11	0.95 ± 0.06	< 0.0001
Liver weight (g)	18.16 ± 1.9	7.20 ± 0.64	< 0.0001

^a^Two-tailed Mann–Whitney test

**Table 2:** Urinary creatinine-corrected amino acids excretion (µM/mM) in the L-ZSF1 and O-ZSF1 rats at the age of 20 weeks, and absolute and percentage difference between the two groups

Amino acids, PTM, AGEs	O-ZSF1 (*n* = 12)	L-ZSF1 (*n* = 12)	*P* value^a^	Difference (%)^b^
Alanine (Ala)	27.4 [15.8–45.4]	21.4 [17.7–24.2]	0.657	+ 6.0 (22)
Threonine (Thr)	48.6 [28.6–75.8]	32.0 [23.1–40.8]	0.028	− 16.6 (34)
Glycine (Gly)	89.9 [73.3–157]	115.5 [82.8–139.5]	0.688	− 25.6 (22)
Valine (Val)	11.3 [8.28–24.2]	6.75 [4.47–13.1]	0.052	+ 4.55 (40)
Serine (Ser)	39.8 [25.1–72.8]	42.2 [29.5–45.3]	0.300	− 2.4 (6)
Leucine + Isoleucine (Leu + Ile)^c^	19.9 [13.4–33.4]	16.6 [9.31–26.7]	0.319	+ 3.3 (17)
Asparagine + Aspartate (Asn + Asp)^c^	29.6 [18.9–54.4]	36.9 [24.9–39.8]	0.641	− 7.3 (20)
Proline (Pro)	1.36 [0.93–1.94]	0.80 [0.69–1.11]	0.009	+ 0.56 (41)
Glutamine + Glutamate (Gln + Glu)^c^	146 [121–348]	95.0 [68.5–139.5]	0.014	+ 51 (35)
Ornithine + Citrulline (Orn + Cit)^c^	8.67 [5.74–11.9]	6.54 [4.64–7.42]	0.084	+ 2.13 (25)
Phenylalanine (Phe)	4.81 [3.12–7.52]	4.15 [2.51–5.52]	0.410	+ 0.66 (16)
Tyrosine (Tyr)	4.27 [2.83–7.02]	3.19 [2.41–3.88]	0.193	+ 1.08 (25)
Lysine (Lys)	21.1 [12.8–36.2]	23.7 [15.6–38.2]	0.842	− 2.6 (11)
Arginine (Arg)	8.40 [5.16–11.2]	6.81 [5.95–10.7]	0.945	+ 1.59 (19)
Tryptophan (Trp)	1.06 [0.66–1.82]	0.75 [0.59–0.96]	0.272	+ 0.31 (29)
Sarcosine (Sarc)	0.24 [0.17–0.5]	0.19 [0.14–0.22]	0.024	+ 0.05 (21)
Guanidinoacetate (GAA)	27.0 [21.8–30.2]	31.4 [23.2–37.6]	0.395	− 4.4 (14)
Homoarginine (hArg)	0.06 [0.03–0.108]	0.11 [0.08–0.15]	0.025	− 0.05 (45)
Asymmetric dimethylarginine (ADMA)	0.175 [0.15–0.275]	0.195 [0.13–0.25]	0.989	− 0.025 (13)
Dimethylamine (DMA)	75.2 [53.4–93.3]	68.2 [56.5–102.9]	0.496	+ 7 (9)
Monomethyl-arginine (MMA)	0.09 [0.05–0.14]	0.08 [0.05–0.1]	0.668	+ 0.01 (11)
Monomethyl-lysine (MMK)	0.11 [0.073–0.13]	0.065 [0.06–0.078]	0.013	+ 0.045 (41)
4-Hydroxy-proline (OH-Pro)	0.38 [0.285–0.778]	0.28 [0.23–0.358]	0.110	+ 0.1 (26)
5-Hydroxy-lysine (D) (5-HO-K-D)	0.92 [0.80–1.21]	0.63 [0.55–0.81]	0.049	+ 0.29 (32)
5-Hydroxy-lysine (L) (5-HO-K-L)	3.51 [2.66–4.62]	3.02 [2.52–3.82]	0.326	+ 0.49 (14)
Carboxymethyl-lysine (CML)	1.82 [1.12–3.30]	1.37 [0.91–1.63]	0.139	+ 0.45 (25)
Carboxyethyl-lysine (CEL)	0.54 [0.43–0.99]	0.29 [0.212–0.338]	0.0007	+ 0.25 (46)
Furosine (*N*^ε^-(2-furoylmethyl)-lysine)	0.38 [0.24–0.44]	0.25 [0.18–0.31]	0.139	+ 0.13 (34)
Carboxyethyl-arginine (CEA)	0.80 [0.5–1.12]	0.67 [0.437–0.715]	0.355	+ 0.13 (16)
Carboxyethyl-cysteine (CEC)	1.71 [1.15–2.34]	0.94 [0.71–1.22]	0.0047	+ 0.77 (45)
Succinyl-cysteine (2SC)	2.89 [2.00–3.52]	2.30 [2.01–2.73]	0.172	+ 0.59 (20)

^a^Two-tailed Mann–Whitney test

^b^Absolute and percentage difference between O-ZSF1 and L-ZSF1 rats

^c^For these pairs of amino acids only their sum can be provided because of the GC–MS method (Hanff et al. 2019)

Statistically significantly different creatinine-corrected urinary excretion rates between the L-ZSF1 and O-ZSF1 rats were observed for Thr, Pro, Gln + Glu, Sarc, hArg, MMK, 5-OH-K-D, CEL, and CEC. Among the native amino acids, solely the excretion rate of hArg was lower (− 45%) in the O-ZSF1 compared to the L-ZSF1 rats (Table 2). In humans, hArg is a metabolite of Lys (Bollenbach et al. 2019). In our study, there was a close correlation between the urinary concentrations of Lys and hArg both in the O-ZSF1 (*r* = 0.931, *P* = 3.9 × 10^–5^) and in the L-ZSF1 (*r* = 0.986, *P* = 1.7 × 10^–7^) rats.

The serum glucose concentration correlated with the urinary creatinine concentration in the L-ZSF1 rats (*r* = 0.622, *P* = 0.035), but not in the O-ZFS1 rats (*r* = − 0.200, *P* = 0.530). The serum glucose concentration did not correlate with any urinary metabolite in the O-ZSF1 rats. In the L-ZSF1 rats, however, the serum glucose concentration did correlate inversely with urinary Ala (*r* = − 0.643, *P* = 0.028), and inversely borderline with 2SC (*r* = − 0.538, *P* = 0.075).

**Fig. 2:**
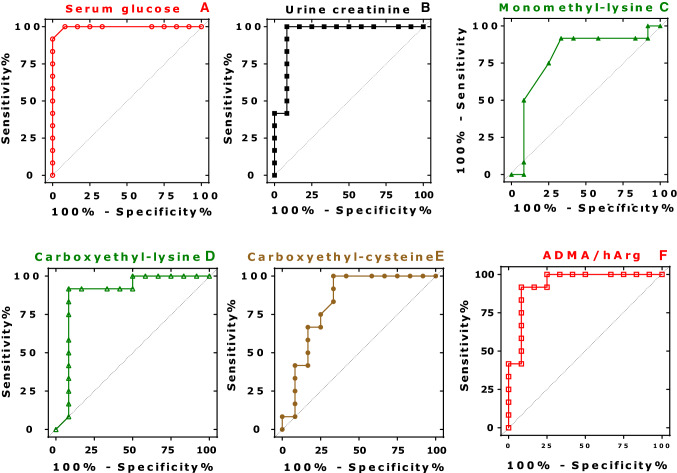
AUROC curve diagrams for serum glucose concentration (**A**), urinary creatinine concentration (**B**), and for urinary creatinine-corrected excretion rates of monomethyl-lysine (**C**), carboxyethyl-lysine (**D**), carboxyethyl-cysteine (**E**), and of the molar ratio ADMA-to-hArg (ADMA/hArg) in O-ZSF1 and L-ZSF1 rats (each *n* = 12)

The results from the ROC analyses of serum and urinary biochemical parameters of the two rat groups are summarized in Table 3. Selected graphs of the ROC curves are presented in Fig. 2. Expectedly, the highest AUROC curve value was obtained for serum glucose concentration (0.996, *P* < 0.0001; Fig. 2A). The AUROC curve values of the serum MDA concentration (0.939, *P* = 0.0004) was also high (Table 3). The urine–creatinine, but not the serum–creatinine, concentration was associated with a high AUROC curve value (0.951, *P* = 0.0002; Fig. 2B).

**Table 3:** AUROC curve and *P* values of the creatinine-corrected urinary excretion rates of the listed analytes, urinary creatinine and serum concentrations between O-ZSF1 and L-ZSF1 rats

Amino acids and metabolites	AUROC curve ± SE	*P* value
Urine		
Ala	0.639 ± 0.121	0.248
Thr	0.708 ± 0.111	0.083
Gly	0.576 ± 0.126	0.525
Val	0.736 ± 0.106	0.049
Ser	0.556 ± 0.130	0.644
Leu + Ile	0.625 ± 0.117	0.298
Asn + Asp	0.538 ± 0.130	0.751
Pro	0.806 ± 0.095	0.011
Gln + Glu	0.792 ± 0.095	0.015
Orn + Cit	0.688 ± 0.114	0.119
Phe	0.604 ± 0.119	0.387
Tyr	0.660 ± 0.114	0.184
Lys	0.542 ± 0.121	0.729
Arg	0.510 ± 0.124	0.931
Try	0.634 ± 0.119	0.260
Sarc	0.722 ± 0.110	0.065
GAA (guanidinoacetate)	0.639 ± 0.120	0.248
hArg	0.750 ± 0.101	0.038
GAA/hArg	0.715 ± 0.107	0.073
ADMA	0.504 ± 0.123	0.977
ADMA/hArg	0.938 ± 0.052	0.0003
DMA	0.576 ± 0.121	0.525
Monomethyl-arginine (MMA)	0.556 ± 0.122	0.644
Monomethyl-lysine (MMK)	0.792 ± 0.103	0.015
4-Hydroxy-proline	0.694 ± 0.111	0.106
5-Hydroxy-lysine (D) (5-OH-Lys-D)	0.736 ± 0.109	0.049
5-Hydroxy-lysine (L) (5-Hydroxy-Lys-L)	0.618 ± 0.119	0.326
Carboxymethyl-lysine (CML)	0.681 ± 0.117	0.133
Carboxyethyl-lysine (CEL)	0.885 ± 0.083	0.0014
Furosine (*N*^*ε*^-(2-furoylmethyl)-lysine)	0.681 ± 0.121	0.133
Carboxyethyl-arginine (CEA)	0.615 ± 0.120	0.341
Carboxyethyl-cysteine (CEC)	0.830 ± 0.090	0.006
*S*-(2-Succinyl)-cysteine (2SC)	0.653 ± 0.118	0.204
Creatinine	0.951 ± 0.049	0.0002
Serum		
Glucose	0.996 ± 0.007	< 0.0001
MDA	0.939 ± 0.048	0.0004
Creatinine	0.674 ± 0.118	0.1569

In urine, the highest AUROC curve values among the non-modified amino acids were observed for Pro (0.806, *P* = 0.011), Gln + Glu (0.792, *P* = 0.015) and Val (0.736, *P* = 0.049). The AUROC curve values of hArg (0.750, *P* = 0.038), especially of its molar ratio to ADMA (ADMA/hArg, 0.938, *P* = 0.0003, Fig. 2F) were statistically significant. Among the PTM metabolites, MMK (0.792, *P* = 0.015; Fig. 2C) and 5-HO-K-D (0.736, *P* = 0.049) led to significant values. With respect to the AGEs analyzed in the present study, statistically significant AUROC curve values were obtained for CEL (0.885, *P* = 0.0014; Fig. 2D) and CEC (0.830, *P* = 0.006; Fig. 2E).

## Discussion

Animal models including those of human diseases, such as diabetes and HFpEF, are useful to study underlying mechanisms of disease development, progression and therapy (Conceição et al. 2016). O-ZSF1 rats spontaneously develop HFpEF, characterized by higher brain natriuretic peptide (BNP), diastolic dysfunction and normal LV-EF% because of obesity, diabetes and hypertension (Valero-Muñoz et al. 2017). In the present study, we measured amino acids and their PTMs and AGEs in urine of 20-week-old O-ZSF1 rats and L-ZSF1 rats, the latter serving as controls.

To test these markers for their diagnostic ability in HFpEF, we prepared ROC analyses. The highest AUROC curve value (0.996) was observed for the serum glucose concentration, which was on average 1.39 times higher in the O-ZFS1 rats compared to the L-ZSF1 rats. This finding confirms the hyperglycemic status of the O-ZFS1 rats. The second-largest AUROC curve value (0.951) was observed for the urinary creatinine concentration, which was 2.5 times lower in the O-ZFS1, likely due to the higher excreted urine volumes of the diabetic rats. The creatinine-corrected excretion rates of Thr, Pro and Gln + Glu were higher in the O-ZSF1 rats. The highest AUROC curve values among the non-modified amino acids were observed for Gln + Glu (0.792) and Pro (0.806), the latter being biosynthesized from l-glutamate (Glu). 4-OH-Pro is a major PTM metabolite of Pro hydroxylation by prolyl-hydroxylase (Li and Wu 2018) and is abundantly present in collagen. In our study, the urinary excretion of 4-OH-Pro did not differ between the O-ZFS1 and L-ZFS1 rats, suggesting no particular role of prolyl-hydroxylase in the HFpEF model.

In the present study, no differences in urine concentrations were found between the O-ZFS1 and L-ZFS1 rats with respect to Arg, MMA, ADMA and its major metabolite DMA (Tsikas 2020), suggesting no significant role of asymmetric dimethylation of Arg residues in proteins in the HFpEF model. The creatinine-corrected hArg excretion rate was almost two times lower in the O-ZSF1 than in the L-ZSF1 rats. In the vasculature, hArg has been proposed to antagonize the action of ADMA (Tsikas and Kayacelebi 2014). The AUROC curve value for the molar ADMA-to-hArg (ADMA/hArg) ratio was comparable with that of creatinine (0.938 vs. 0.951). As low hArg and ADMA excretion rates are associated with diseases in the renal and cardiovascular systems (Frenay et al. 2015; Said et al. 2019a, b), the lower hArg values in the O-ZSF1 rats may suggest elevated renal and cardiovascular disease (CVD) risks in the O-ZSF1 rats.

In contrast to Arg, the concentrations of the PTM and AGEs metabolites of Lys, i.e., MMK and CEL, and the AGE of Cys, i.e., CEC, were higher in the O-ZSF1 compared to the L-ZSF1 rats. The 1.7-fold higher MMK urinary excretion in the O-ZSF1 rats suggests a higher extent of methylation of Lys residues in proteins in these rats. Among the AGEs measured in the urine of the ZFS1 rats in the present study, the highest AUROC curve values were observed for CEL (0.885) and CEC (0.830). These observations may suggest that in this HFpEF animal model, the *N*^*ε*^-amine group of Lys and the SH group of Cys residues in proteins are easier accessible and chemically more reactive to glycation than Arg residues. Plasma concentrations of AGEs in patients with HFpEF and HFrEF are scare (Willemsen et al. 2012; Paulus and Dal Canto 2018). In HFpEF patients of a large longitudinal cohort, the mean plasma CML concentration was 2.05 µM in the survivors and 2.42 µM in the non-survivors of the study, and higher plasma CML concentration was significantly and independently associated with higher risk for mortality (Willemsen et al. 2012). The median plasma concentration of the soluble form of the AGEs receptor, i.e., sRAGE, was lower in the survivors (2.85 ng/mL) compared to the non-survivors (3.38 ng/mL) (Willemsen et al. 2012), suggesting association of elevated formation of AGEs with CVD and mortality in the HFpEF patients.

The concentrations of PTM metabolites and AGEa were found to be associated with oxidative stress. Superoxide dismutase (SOD) is an important counter-regulatory enzyme in the event of oxidative stress. Glycation of Arg and Lys residues in the SOD active site was found to inactivate or at least alter its activity (Anwar and Younus 2017). In type II diabetes mellitus patients, SOD-1 activity was found to be altered (Tavares et al. 2019). In the context of HFpEF, we suppose that SOD glycation, as a consequence of hyperglycemia, may further contribute to increased oxidative stress and endothelial dysfunction.

**Fig. 3:**
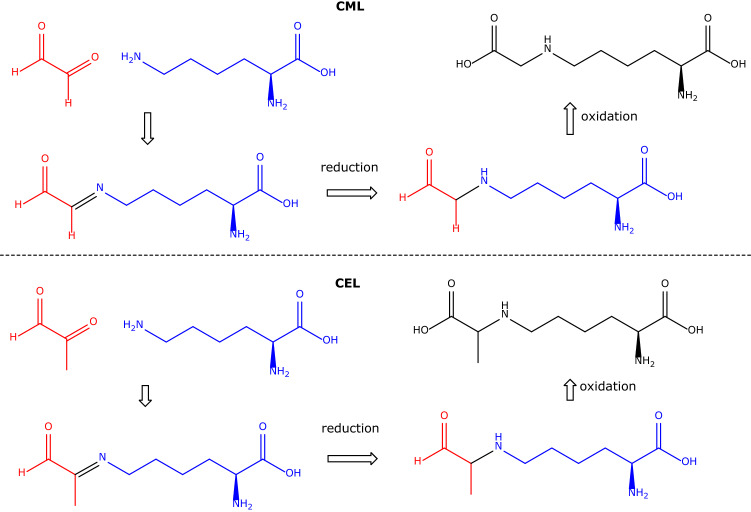
Simplified scheme showing the chemical reaction of glyoxal (CHO-CHO, upper panel) and methylglyoxal (CH_3_–CO–CHO, lower panel) with the terminal *N*^*ε*^-amine group of l-lysine to form CML and CEL, respectively. The Schiff’s bases are reduced and the aldehyde functionalities are oxidized to form the carboxylic groups of CML and CEL

It has been suggested that measurement of CML and CEL in urine may be useful indices of in vivo synthesis of methylglyoxal and glyoxal from glucose metabolism (Ahmed et al. 1997). Other substrates, such as pentoses, ascorbate and glucose, were found to react with Lys residues in proteins such as collagen to form CEL and CML, yet with much lower yields ranging from 0.001% with glucose to 0.26% with methylglyoxal (Ahmed et al. 1997). Such reactions in vitro would require reduction of the Schiff base and oxidation of the remaining aldehyde group (Fig. 3).

In our study, the urinary molar excretion ratio of CML-to-CEL was 3.3 ± 0.9 in the O-ZSF1 group and 5.1 ± 1.6 in the L-ZSF1 group (*P* = 0.0011; two-tailed unpaired *t* test), with the L-ZSF1/O-ZSF1 ratio being 1.7 ± 0.6. Our study suggests that the higher non-enzymatic *N*^*ε*^-glycation of proteinic Lys residues in the O-ZSF1 rats compared to the L-ZSF1 rats may be indicative of an unfavorable glucose metabolism in HFpEF patients.

In human lens proteins, CML and CEL were found to increase with age (Ahmed et al. 1997). As we analyzed rather young animals, it would be of great interest whether advanced age further contributes to CML and CEL level increase. As initially stated, urinary glucose had the highest AUROC curve score what is in line with the hyperglycemic state of O-ZSF1rats. Interestingly, it was shown in human erythrocytes in vitro that the CML and CEL content of globin increases proportionally with glucose addition (Nagai et al. 2005). AGEs are expected to be formed at higher rates in CVD, particularly in diabetes in hyperglycemic conditions (Willemsen et al. 2012; Hanssen et al. 2013), leading to high concentrations in biological systems including blood, tissue and urine. However, in vitro and in vivo studies yielded contradictory and difficult to interpret results. Measurement of CML, CEL, and pentosidine incorporated in plasma of humans with altered and non-altered glucose metabolism of two Dutch cohort studies revealed differences in plasma concentrations of these AGEs (Hanssen et al. 2013). Yet, that study did not find independent adverse associations of the measured plasma AGEs concentrations with CVD in individuals with normal or impaired glucose metabolism including type II diabetes mellitus. Thus, the authors of that study suggested that measurement of AGEs in plasma, as biomarkers for increased CVD risk, may be limited in a population-based setting and proposed the measurement of AGEs and their precursors, for example, free methylglyoxal, in other biological samples such as urine (Hanssen et al. 2013). Concerning our animal model, in which a diabetic state triggers HFpEF manifestation and thus representing an indistinguishable confounder, the study of Hanssen et al. (2013) implies that hyperglycemia may not alone be accountable for altered AGEs concentrations. The usefulness of urinary AGEs as biomarkers in HFpEF should be addressed in clinical studies.

Finally, we also performed analyses in serum for MDA. The serum MDA concentration was higher in the O-ZSF1 rats compared to the L-ZSF1 rats, and the AUROC curve value of MDA was high (0.939, *P* = 0.0004). These observations indicate higher lipid peroxidation in the O-ZSF1 rats presumably due to their hyperlipidemia and hyperglycemia. The high AUROC curve value of serum MDA suggests MDA as a useful biomarker in the HFpEF rat model.

## Study limitations

Findings in animal models of diseases need to be interpreted carefully concerning comparability with and translation to humans. The murine metabolism and the expression profiles of genes and proteins differ from those in humans. As the animals used in our study were in early adolescence at sacrifice, hormone status differs from typical human patient groups. In addition, the experimental procedures may have introduced cardiovascular stress although the animals were in deep sedation. Nevertheless, we assume that, if metabolic alterations were introduced, these should be comparable in both rat groups as they were treated equally. We analyzed rats at an age of 20 weeks when HFpEF is already manifested and progression is at an early stage. It would be meaningful to determine prospectively potential alterations in Arg-involving pathways. O-ZSF1 rats consumed considerably more foods than L-ZSF1 rats. An effect of higher food intake on the urinary excretion of the analytes cannot be excluded, although dietary AGEs including CML by pregnant rats were found to exert no metabolic and renal effects (Janšáková et al. 2019). Urinary creatinine excretion was 2.5-fold higher in the L-ZSF1 rats compared to the O-ZSF1 rats.

## Conclusion

L-ZSF1 and O-ZSF1 rats excrete higher amounts of Lys and Cys metabolites, including MMK, CEL and CEC. Higher urinary excretion rates of MMK, CEL and CEC and higher serum MDA concentrations may be useful biomarkers in the ZSF1 rat model of HFpEF. The O-ZSF1 rats should be useful to investigate potential effects of AGEs, such as CEL and CEC in HFpEF.

## Abbreviations

ADMA: Asymmetric dimethylarginine
AGEs: Advanced glycation end-products
AUROC: Area under (AU) the receiver operating characteristic (ROC)
CVD: Cardiovascular disease
DMA: Dimethylamine
HFpEF: Heart failure with preserved ejection fraction
HFrEF: Heart failure with reduced ejection fraction
MDA: Malondialdehyde
MMK: Monomethyllysine
PTM: Post-translational modifications
ROC: Receiver operating characteristic
SOD: Superoxide dismutase
sRAGE: Soluble form of AGEs receptor
ZSF1: Cross-breed between a **Z**ucker Diabetic Fatty (ZDF) female rat and a **S**pontaneously hypertensive heart failure (SHHF) male rat in **F1** generation
